# Report on Influenza A and B Viruses: Their Coinfection in a Saudi Leukemia Patient

**DOI:** 10.1155/2013/290609

**Published:** 2013-09-02

**Authors:** Fahad N. Almajhdi, Ghazanfar Ali

**Affiliations:** ^1^Department of Botany and Microbiology, College of Science, King Saud University, P.O. Box 2455, Riyadh 11451, Saudi Arabia; ^2^Center of Excellence in Biotechnology Research, King Saud University, P.O. Box 2460, Riyadh 11451, Saudi Arabia; ^3^Department of Biotechnology, University of Azad Jammu and Kashmir, P.O. Box 13100, Muzaffarabad, Pakistan

## Abstract

*Purpose*. Influenza A and B viruses are the leading cause of respiratory infections in children worldwide, particularly in developing countries. There is a lack of data on coinfection of influenza A and B viruses circulating in Saudi Arabia. In this study, we aimed to identify the circulation of influenza viruses that contribute to respiratory tract infections in Saudi children. *Methods*. We collected 80 nasopharyngeal aspirates (NPAs) from hospitalized children with acute respiratory illness (ARI) at Riyadh during the period extended from October 2010 till April 2011. Samples were tested for the common respiratory viruses including influenza viruses by RT-PCR. *Results*. Overall, 6 samples were found positive for influenza A and/or B viruses. Among these positive clinical samples, only one collected sample from a female one-year-old immunocompromised child with leukemia showed a coinfection with influenza A and B viruses. In present study coinfection was confirmed by inoculation of the clinical specimen in specific pathogenfree embryonating chicken eggs and identification of the virus isolates by hemagglutination and one-step RT-PCR. *Conclusion*. This study opens the scene for studying the role of influenza virus's coinfection in disease severity and virus evolution. Further studies are required to better understand the clinical importance of viral coinfection.

## 1. Introduction

Influenza viruses are the most important infectious agents of childhood and thus highly prone to contribute to feverish episodes in children with cancer [1, 2]. Majority of the patients with influenza infections show flu-like symptoms with a benign course, while patients with comorbidities may have a serious clinical appearance with respiratory failure [3]. Influenza viruses are more allied with considerable morbidity and mortality in cancer patients, and several million people were infected worldwide [4]. The most important cause of death in all immunocompromised populations is infection; however, 35–80% of patients with leukemia and influenza develop influenza pneumonia, and around 43% of patients having leukemia and influenza pneumonia die because of infection [5, 6]. Immunosuppressed patients such as those receiving chemotherapy for leukemia, are at increased threat of contracting influenza viruses, together with the possibility of more severe infection [7, 8]. Annual epidemics of influenza illness are responsible for considerable economic losses as a result of hospitalization, medication costs, work and school absence including mortalities.

Coinfection of influenza A and B viruses is a rare incident [9]. Coinfection has not been entirely explored due to some limitations of many studies. Some studies focused on younger age groups, hospitalized patients, or deceased individuals, which do not symbolize the general population; similarly, some others have utilized a small sample size or limited their focus to certain viral pathogens, underestimating the role of other viruses in coinfection [10]. Such coinfections have not been extensively described or characterized as a consequence of the prior well-known use of culture-based methods for identification. Just few authors have published simultaneous infection by two disparate strains of influenza viruses in human beings [11, 12]. Medical manifestations in these cases of influenza with dual infections were the same as those observed in single infections. The epidemiological and virological study of dual influenza infection cases in humans is of major interest particularly for the monitoring of newly emerging influenza strains, which could perpetuate epidemic or pandemic events.

In our study, influenza viruses has been identified in Riyadh, Saudi Arabia, circulating during the 2010-2011 season, as in other parts of the world by a significant cocirculation of influenza A and influenza B viruses [13]. In this paper, we present a single case of coinfection caused by influenza A and B viruses in immunocompromised patient. The major endeavor of this study was to investigate prospectively the fate of viral infections (single and coinfection) and their cocirculation in children with an acute respiratory illness applying RT-PCR techniques of nasopharyngeal aspirates (NPAs). 

## 2. Methods

### 2.1. Samples Collection

From October 2010 to April 2011, 80 nasopharyngeal aspirates (NPAs) were collected from children hospitalized with acute respiratory illness at three different hospitals located in Riyadh province, Saudi Arabia. All patients presented typical clinical symptoms of influenza, including sore throat, cough, and fever >39°C. Other symptoms like nasal congestion and diarrhea were reported only in few patients. Sample collection was approved by the Ethical Committee of King Saud University. All the aspirates were taken from patients, after getting an informed consent from their parents or guardians. Each aspirate was placed immediately in 2 mL viral transport medium containing minimal essential medium (MEM), 1,000 U penicillin, and 1 mg streptomycin and shipped on dry ice to the Virology Research Laboratory, College of Science, King Saud University. Upon arrival, the aspirates were vortexed for 15 sec and divided into aliquots (140 *μ*L each). One aliquot was used for viral RNA extraction, while the rest was stored at −80°C.

### 2.2. RNA Extraction

Extraction of viral RNA from clinical samples was conducted using viral RNA extraction kit (Qiagen, Hilden, Germany) according to the manufacturer's guidelines. Each clinical sample aliquot was used only once to avoid the loss of viral RNA by multiple freezing and thawing. 140 *μ*L sample volumes were processed to obtain 60 *μ*L RNA elutes. Five microliters of the extracted RNA were used immediately in RT-PCR, while the rest was splitted into aliquots and kept frozen at −80°C for further analysis.

### 2.3. RT-PCR for Detection of Influenza Viruses

Detection of influenza A and B viruses in clinical samples was accomplished by simultaneous amplification of specific sequences located within the NS-2 gene of either virus using the primer sets (Table 1) designed originally by Claas et al. [14]. The RT-PCR reaction was conducted using one-step RT-PCR kit (Qiagen) according to the manufacturer's instructions. The reaction tubes were incubated in the thermal cycler Gene-Amp 9700 (Applied Biosystems, Foster City, CA, USA) for one cycle of reverse transcription at 50°C for 30 min; one cycle of initial PCR activation at 95°C for 15 min; 35 cycles of denaturation at 94°C for 30 sec, primer annealing at 50°C (for influenza A) and 56°C (for influenza B) for 30 sec, and extension at 72°C for 1 min; and a final extension step at 72°C for 10 min. In each RT-PCR assay positive and negative controls were included. PCR products were resolved in 1.5% agarose gel containing ethidium bromide (0.5 *μ*g/mL); the product bands were identified in comparison with 100 bp DNA ladder (Qiagen) and documented using a gel documentation system (IMAGO compact imaging system, B&L, USA).

### 2.4. Isolation of Influenza Viruses Using Embryonating Chicken Eggs

Aliquots of the NPAs, that showed positive results in RT-PCR, were diluted in serum-free MEM containing 20 mg/mL pancreatic trypsin (Sigma; 50% v/v). Approximately 0.2 mL of the clinical sample was inoculated in 9–11 days old specific pathogen-free (SPF) embryonating chicken eggs. Eggs were incubated in a stationary incubator at 37°C for 4–7 days and were candled daily for embryonic viability. Eggs containing dying embryos after 24 hours of incubation and those who stayed alive till the end of incubation period were chilled for two hours, and the allantoic fluid was harvested aseptically. Three successive passages were performed for complete adaptation and propagation of the virus isolates. The virus identity and titer were confirmed by plate haemagglutination assay [15]. One-step RT-PCR assay was performed using viral RNA extracted from allantoic fluids to confirm isolation of influenza A and/or B viruses. 

## 3. Results

**Figure 1:**
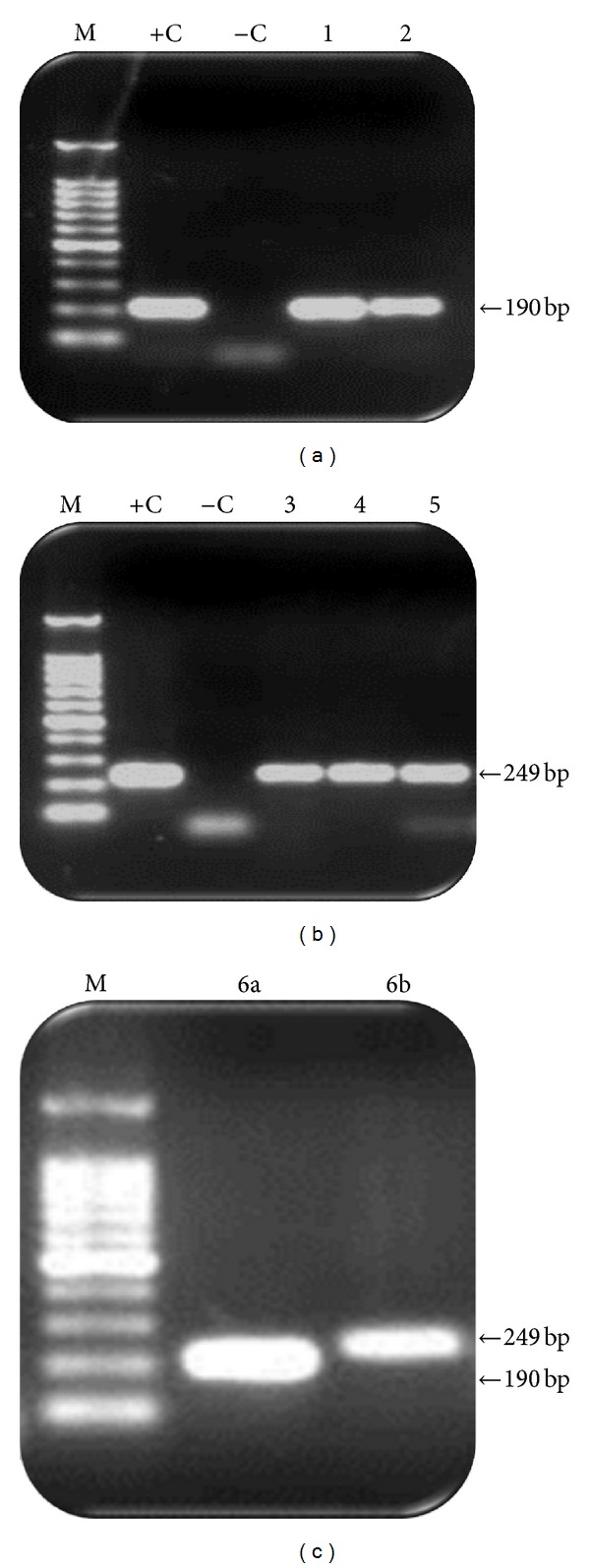
((a), (b), (c)) Ethidium bromide stained 1.2% agarose gel showing reverse transcription polymerase chain reaction (RT-PCR) products of influenza A and B viruses. (a) One-step RT-PCR (influenza A, gene NS-2): lane M, 100 bp DNA marker; lane +C, positive control influenza A; lane −C, negative control; lane 1, influenza A sample 1; lane 2, sample 2. (b) One-step RT-PCR (Influenza B, gene NS-2): lane M, 100 bp DNA marker; lane +C, positive control influenza B; lane −C, negative control; lane 1, influenza B sample 3; lane 2, sample 4; lane 3, sample 5. (c) One-step RT-PCR (influenza A and B): lane M, 100 bp DNA marker; lane 1, influenza A sample 6a; lane 2, influenza B sample 6b.

In the present study, one-step RT-PCR assay was performed for detection of influenza A and B viruses using viral RNA extracted from 80 NPAs of hospitalized children at Riyadh during the period October 2010–April 2011. Using RT-PCR as the gold standard technique, the results showed that 6 (7.5%) samples were positive for influenza viruses (Figures 1(a) and 1(b)), among which only one sample (1.25%) was positive for both types of influenza viruses (Figure 1(c)) as a coinfection. The coinfection was further confirmed by virus isolation in SPF embryonating chicken eggs.

The sole sample that included both influenza A and B viruses was identified in a one-year-old female leukemia patient without any history of influenza vaccination coverage. At the time of inclusion and sampling, she was presenting typical influenza-like disease symptoms including fever >39°C, myalgia, pharyngitis, and cough. She had not travelled out of Saudi Arabia during 2010–2011 winter season. 

## 4. Discussion

In the present study, influenza A and B viral RNA were simultaneously detected in a single NPA of immunocompromised patient. To the best of our knowledge, this is the first report on coinfection of influenza A and B viruses in Saudi Arabia. In this study, influenza virus's infection in leukemia patient was associated with mild symptoms similar to those of seasonal influenza-like illness including fever, cough, and sore throat. It is more remarkable to note that co-infected patient reported in this study represented with a weak immune system, being a one-year-old leukemia patient, thus suggesting that, in fact, immunological state of the patient may play an important role in the establishment of coinfection. In the literature, very little information is accessible concerning the frequency of respiratory viruses in immunocompromised patients of the Middle East region. As recommended by our virological data records, the detection of coinfections had most likely been underestimated maybe due to lack of sensitivity of conventional methods, including quick antigen detection assays and conventional rapid cell culture assays [16, 17]. 

In our cohort, we detected six cases of influenza viruses including a single case of dual infection by influenza A and B viruses. Coinfections of influenza A and B viruses come out to be infrequent event, and only few authors have reported simultaneous infection by two different types of influenza viruses in human being [9, 12, 18]. In previous studies, authors have presented that in case of coinfection caused by influenza type A and B viruses, the titers of influenza A viruses were ten thousand times higher than those of influenza B viruses in the clinical samples [11, 12]. The factors that may be accountable for such event are not clear yet, although the host immune system and the virus properties have been suggested [19]. To date, the mechanism of coinfection is unclear; we consider that instances of coinfections should be taken critically by both clinicians and researchers.

In this study report, only single leukemia patient with a coinfection presented with typical clinical symptoms of influenza-like illness. Moreover, this study recommended that influenza coinfection in patients was not linked with the severity of the symptoms [20]. Some past studies have established that the coinfections by influenza A and human respiratory syncytial viruses were not allied with more severe signs than monoinfections in the children [21]. Most of the published data have analyzed the recurrent trends and clinical patterns of respiratory viral infections in neonates and immunocompetent children of developing countries [22].

In the current study, coinfection by two influenza viruses in humans appeared as an exceptional event. However, influenza coinfections correspond to a possible source of multiple viral transmissions and comprise a basis for viral recombination events between two human strains or between a human and an avian viral strain [23]. Therefore, it may possibly be of major interest to develop faster antigen detection assays or RT-PCR assays for the identification of dual infections by influenza viruses during the winter epidemic season. Most of the prior studies including this current study put forward that the risks of influenza diseases are especially high among severely immunosuppressed patients. Unfortunately, these immunosuppressed leukemia patients are less possible to mount an ample of antibody response to active immunization [24, 25].

It is particularly important to recognize influenza in hospitalized patients so that proper infection control procedures can be implemented more rapidly. Due to high morbidity and mortality associated with influenza diseases in leukemia patients, efficient prophylactic and curative regimens need to be defined. 

All the PCR-based molecular diagnostic methods are effective utensils that enable researchers to broadly characterize the circulating strains of influenza viruses, including dual infections, without the use of culture-based classical methods. Despite these facts, our results suggest that increase in awareness of potentially lethal outcomes in patients co-infected with influenza A and B viruses is needed from the clinical and public health community to develop prevention and control strategies commensurate with the importance of these pathogens. Further studies concerning antigenic and genomic characterization of the influenza viruses, as well as the basis of their diversity and evolution, are in progress and also expected to provide valuable information.

The major aim of our study was detection of circulating influenza viruses and their coinfection. The incidence of more than two viruses in the same sample may not always show clinical infection. Previous studies [10] suggest coinfection may manifest higher disease severity, which may shorten the time to medical care and viral detection; disease severity was not assessed in the current study. Our findings from current study show that in case of hospitalized patients with respiratory tract infection, especially immunocompromised patients who are susceptible to serious complications, the simultaneous detection of multiple viral agents is advisable and more reliable than detection of only single viruses. Our data is very limited, so further studies are needed in pediatric oncology cases for the detection of coinfection by influenza/respiratory viruses in hospitalized Saudi patients.

## Figures and Tables

**Table 1:** Primers used in this study.

Virus	Primer name	Primer sequence (5′→3′)	PCR product
Influenza A virus	INF-A-F1	AAGGGCTTTCACCGAAGAGG	190 bp
	INF-A-R1	CCCATTCTCATTACTGCTTC	
Influenza B virus	INF-B-F1	ATGGCCATCGGATCCTCAAC	249 bp
	INF-B-R1	TGTCAGCTATTATGGAGCTG	
